# Interplay between Inflammation and Stemness in Cancer Cells: The Role of Toll-Like Receptor Signaling

**DOI:** 10.1155/2016/4368101

**Published:** 2016-12-27

**Authors:** Da-Wei Yeh, Li-Rung Huang, Ya-Wen Chen, Chi-Ying F. Huang, Tsung-Hsien Chuang

**Affiliations:** ^1^Immunology Research Center, National Health Research Institutes, Miaoli 35053, Taiwan; ^2^Institute of Molecular and Genomic Medicine, National Health Research Institutes, Miaoli 35053, Taiwan; ^3^National Institute of Cancer Research, National Health Research Institutes, Miaoli 35053, Taiwan; ^4^Institute of Biopharmaceutical Sciences, National Yang-Ming University, Taipei 11221, Taiwan; ^5^Program in Environmental and Occupational Medicine, Kaohsiung Medical University, Kaohsiung 807, Taiwan

## Abstract

Cancer stem cells (CSCs) are a small population of cancer cells that exhibit stemness. These cells contribute to cancer metastasis, treatment resistance, and relapse following therapy; therefore, they may cause malignancy and reduce the success of cancer treatment. Nuclear factor kappa B- (NF-*κ*B-) mediated inflammatory responses increase stemness in cancer cells, and CSCs constitutively exhibit higher NF-*κ*B activation, which in turn increases their stemness. These opposite effects form a positive feedback loop that further amplifies inflammation and stemness in cancer cells, thereby expanding CSC populations in the tumor. Toll-like receptors (TLRs) activate NF-*κ*B-mediated inflammatory responses when stimulated by carcinogenic microbes and endogenous molecules released from cells killed during cancer treatment. NF-*κ*B activation by extrinsic TLR ligands increases stemness in cancer cells. Moreover, it was recently shown that increased NF-*κ*B activity and inflammatory responses in CSCs may be caused by altered TLR signaling during the enrichment of stemness in cancer cells. Thus, the activation of TLR signaling by extrinsic and intrinsic factors drives a positive interplay between inflammation and stemness in cancer cells.

## 1. Introduction

Inflammation is a hallmark of cancer development [1, 2]. Chronic inflammation caused by exposure to environmental agents, infection, genetic disease, and metabolic disorders is closely associated with several tumors, including lung carcinoma, hepatocellular carcinoma (HCC), gastric cancer, cervical cancer, colorectal cancer, Hodgkin's disease, and multiple myelomas [3, 4].

The tumor microenvironment comprises cancer cells, cancer stem cells (CSCs), endothelial cells, and immune cells, such as tumor-associated macrophages, tumor-associated neutrophils, lymphocytes, and other stromal cells, such as cancer-associated fibroblasts [2, 5]. Inflammatory stimuli, such as the toll-like receptor (TLR) agonists, tumor necrosis factor- (TNF-) *α*, and interleukin- (IL-) 1, activate NF-*κ*B in tumor cells [6, 7]. Activated NF-*κ*B induces the transcription of multiple proinflammatory genes and is a key mediator of acute and chronic inflammatory responses in tumor cells and the tumor microenvironment [3, 8]. NF-*κ*B-mediated proinflammatory programs link cancer-related inflammation with carcinogenic processes, including tumor initiation, tumor promotion, and metastasis. NF-*κ*B induces the expression of genes that regulate apoptosis, angiogenesis, proliferation, survival, and cancer cell invasion. These NF-*κ*B-mediated responses also rehabilitate the inflammatory tumor microenvironment, further supporting tumor progression [9–11].

CSCs are a small population of cancer cells with enriched stemness and tumor-initiating ability in the tumor microenvironment. CSCs possess self-renewal and differentiation abilities, which promote tumor progression and metastasis and are responsible for treatment resistance and cancer relapse [12–16]. In addition to supporting angiogenesis, proliferation, and cancer cell survival, NF-*κ*B-mediated inflammatory responses also support CSC expansion. NF-*κ*B activation induces the expression of stemness-associated genes and regulators of the epithelial-mesenchymal transition (EMT) in cancer cells, thereby generating a CSC phenotype [17–19]. In contrast, together with increased stemness, CSCs exhibit an elevated expression of inflammatory genes due to elevated NF-*κ*B activation [20–23]. This interplay between inflammation and stemness could enhance these two properties of cancer cells to further expand the CSC population. CSCs may cause malignancy and reduce the success of cancer treatments; therefore, the molecular mechanisms underlying the interplay between inflammation and stemness should be further investigated in cancer cells.

TLRs are a family of receptors that sense pathogen-associated molecular patterns (PAMPs) of microbes and danger-associated molecular patterns (DAMPs) released from damaged tissues or cells killed during different cancer therapies [24–26]. TLR signaling triggers NF-*κ*B activation and inflammatory responses [27, 28]. In this review, we discuss the current knowledge on the function and mechanisms of TLR signaling in the bidirectional interplay between inflammation and stemness in cancer cells.

## 2. TLRs

Toll is a type I transmembrane receptor that was originally identified in* Drosophila* for its involvement in embryo development [29]. In the adult fly, toll plays a crucial role in innate immune responses to microbial infections. Thirteen TLRs have been identified in mammals, 10 of which (TLR1–TLR10) are expressed in humans. The 10 human TLRs share common structural features: an extracellular domain comprising multiple leucine-rich repeats, a transmembrane region, and a highly conserved cytoplasmic toll/IL-1 receptor (TIR) domain [30–32].

The cellular location and ligands of these TLRs are summarized in Table 1. TLR3, TLR7, TLR8, and TLR9 are localized in intracellular vesicles, including endosomes, whereas others are localized on the cell surface. These TLRs play essential roles in the innate recognition of PAMPs of microbes. TLR2 recognizes a broad range of microbial components, including peptidoglycan, lipoteichoic acids, lipoproteins, lipoarabinomannan, glycophosphatidylinositol anchors, porins, and zymosan [33–39]. TLR2 can form heterodimers with TLR1 or TLR6 to differentially recognize different microbial products. The TLR2–TLR6 complex preferentially recognizes mycoplasma macrophage-activating lipopeptide 2, whereas the TLR2-TLR1 heterodimer more specifically recognizes bacterial lipoproteins and triacyl lipopeptides [40–42]. TLR3 recognizes double-stranded RNA (dsRNA), which is generated during viral replication within infected cells [43]. TLR4 was the first mammalian TLR to be identified [44] and is the major receptor involved in recognizing lipopolysaccharides on the outer membrane of gram-negative bacteria [45]. TLR5 recognizes flagellin, a component of bacterial flagella [46]. TLR7–TLR9 comprised a TLR subfamily with members containing longer extracellular domains [47, 48]. TLR7 and TLR8 recognize single-stranded RNA viruses, such as the vesicular stomatitis virus or the influenza virus [49, 50]. TLR9 is essential for the response to microbial unmethylated CpG DNA. Most CpG sites in mammalian cells, but not in microbes, are methylated; therefore, unmethylated CpG DNA may indicate a microbe infection [51, 52]. The natural ligand of TLR10 has not yet been identified.

TLRs also recognize a wide variety of endogenous ligands released from damaged tissues or cells killed during different cancer treatments (Table 1). These endogenous ligands are called DAMPs because they are released following tissue injury and cell death and serve as alarmins to trigger TLR activation, thereby providing an early warning signal to the immune system. DAMPs can be cellular components or stress-induced gene products, including extracellular matrix components, extracellular proteins, intracellular proteins, and nucleic acids [53, 54]. TLR2 and TLR4 recognize more DAMPs than other TLRs. TLR2 recognizes heat shock proteins (HSPs), Gp96 biglycan, hyaluronic acid, hyaluronan, HMGB1, versican, and monosodium urate crystal [55–63]. TLR4 senses HSPs, Gp96, HMGB1, oxidized phospholipids, heparin sulfate, fibrinogen, fibronectin, tenascin-C, *β*-defensin 2, hyaluronic acid, and hyaluronan [56, 61–71]. TLR3, TLR7, and TLR8 are activated by host RNA, and TLR9 is activated by host DNA from necrotic cells under special conditions, such as the formation of HMGB1, LL37, or immunoglobulin complex to facilitate ligand interaction with TLR [72–75].

## 3. TLR Signaling

**Figure 1:**
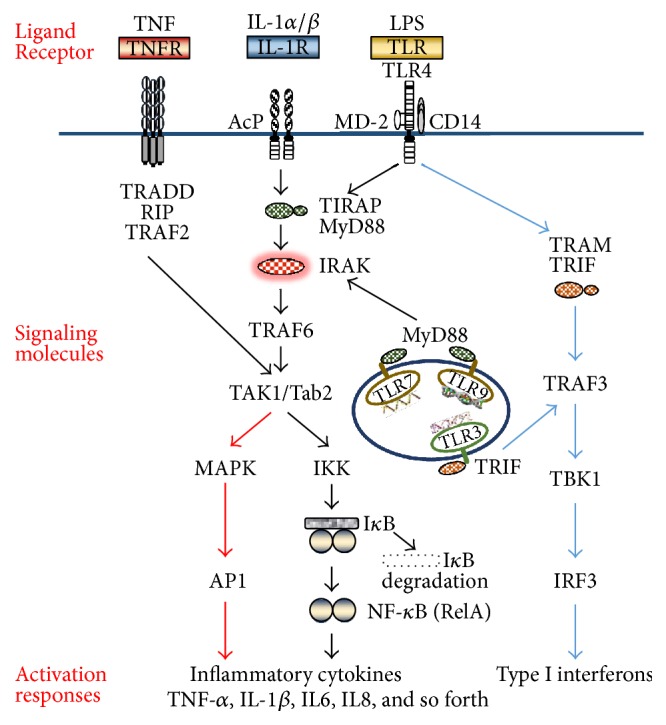
TLR and the related TNFR and IL-1R signaling pathways. TLRs utilize a MYD88 dependent pathway (black line) and a TRIF dependent pathway (blue line) to activate NF-*κ*B, AP-1, and IRF3, leading to the production of inflammatory cytokines and type I interferons. IL-1R uses the same set of signaling molecules, and TNFR utilizes the same signaling pathway as TLRs. TLR, toll-like receptor; IL-1R, interleukin-1 receptor; TNFR, tumor necrosis factor receptor; LPS, lipopolysaccharide; TRADD, TNFRSF1A associated via death domain; TIRAP, TIR domain containing adaptor protein; RIP, receptor interacting serine/threonine kinase; IRAK, interleukin-1 receptor associated kinase; TRAF2, TNF receptor associated factor 2; TRAF3, TNF receptor associated factor 3; TRAF6, TNF receptor associated factor 6; TAK1, TGF-beta activated kinase 1; MAPK, mitogen-activated protein kinase 1; IKK, I-kappa B kinase; I*κ*B, NFKB inhibitor; Tab2, TGF-beta activated kinase 1 binding protein 2; TBK1, TANK-binding kinase 1; MYD88, myeloid differentiation primary response 88; TRIF, TIR domain containing adapter-inducing interferon-*β*; NF-*κ*B, nuclear factor kappa-light-chain-enhancer of activated B cells; AP-1, activator protein 1; IRF3, interferon regulatory factor 3.

In general, when the extracellular domain of TLR is bound by its ligand, two TLR monomers bridge to form a dimer. The TLR dimer then recruits adaptor proteins from the MyD88 family to initiate downstream signaling pathways (Table 1 and Figure 1). The MyD88 adaptor protein family contains five members: MyD88, TRIF/TICAM-1, TIRAP/Mal, TIRP/TRAM, and SRAM [76, 77]. All TLRs except TLR3 signal through a MyD88 dependent pathway, in which a MyD88/IRAK1/IRAK4/TRAF6 complex activates TAK1, which leads to the activation of transcription factors, including NF-*κ*B and AP-1 [78–80]. TLR3 and TLR4 utilize a MyD88-independent pathway, recruiting TRIF to activate IRF3/7 and NF-*κ*B. IRF3/7 activation involves TBK1-IKK*ε*/IKKi complex; NF-*κ*B and AP-1 activation involves TRAF6 and RIP [81–83].

IL-1*β* and TNF-*α* are potent proinflammatory cytokines. TLR agonists and these two cytokines are major mediators of inflammation in the tumor microenvironment [6, 7]. As shown in Figure 1, the IL-1 receptor (IL-1R) utilizes the same signal transduction pathway as that utilized by TLRs: the sequential recruitment of MyD88, IRAK, and TRAF6 to form a complex and TAK activation leading to NF-*κ*B activation. NF-*κ*B activation downstream of the TNF-*α* receptor (TNFR) is mediated by TRADD, RIP, and TRAF2. The molecular components involved in TLR/IL-1R and TNFR signaling pathways only partially overlap; nevertheless, the regulation of these pathways is similar and involves the recruitment of adaptor molecules and ubiquitination-mediated regulation of protein expression and interaction.

## 4. Regulation of TLR Signaling

Ubiquitination regulates TLR signaling, leading to NF-*κ*B activation [28, 84]. Ubiquitination is an enzymatic cascade involving three kinds of enzyme: a ubiquitin-activating enzyme (E1), a ubiquitin-conjugating enzyme (E2), and a ubiquitin-protein ligase (E3). Reactions occur with a single ubiquitin (monoubiquitination) or a chain of ubiquitins (polyubiquitination) conjugated to substrates. The specific ubiquitination of signaling molecules is mediated by E3 ligases (E3s) and counteracted by deubiquitinases (DUBs) [85–89]. There are approximately 600 E3s and 100 DUBs encoded in the human genome. E3s are characterized by distinct domains and can be divided into three groups: HECT, RING, and F-box. DUBs comprise five families: ovarian tumor proteases, ubiquitin-specific proteases (USPs), ubiquitin C-terminal hydrolases, Josephines, and JAB1/MPN/MOV34 metalloenzymes [86–89]. Seven internal lysine residues (K6, K11, K27, K29, K33, K48, and K63) and an N-terminal methionine (M1) residue in ubiquitin can be employed to generate eight structurally and functionally different ubiquitin chains. The type of chain depends on which lysine or M1 residue within a target protein/ubiquitin is attached to the C-terminal glycine of the incoming ubiquitin. K48 ubiquitination is involved in protein degradation via a ubiquitin-proteasome dependent pathway and K63 ubiquitination has been linked to protein-protein interaction for signal transduction [85–89]. Depending on the type of ubiquitination and target protein, E3 ubiquitin-protein ligases and DUBs can serve as positive or negative regulators of NF-*κ*B activation following TLR activation.

Tables 2 and 3 list some E3 ubiquitin-protein ligases and DUBs that function as negative regulators of TLR signaling. For example, K48 ubiquitination promotes ubiquitin-proteasome degradation of I*κ*B by the SCF^*β*TrCP^-E3 complex and subsequent NF-*κ*B activation [90, 91]. NF-*κ*B signaling is negatively regulated by USP11 or USP15-mediated removal of K48-linked ubiquitin chains from I*κ*B*α* [92, 93]. Triad3A/RNF216 and SOCS1 regulate K48 ubiquitination and proteasomal degradation of TIRAP [94, 95]. SOCS1, COMMD1, and PDLIM2 catalyze K48-linked polyubiquitination and facilitate proteasomal degradation of p65/RelA [96–100]. The K63-linked ubiquitin chain in RIP, TRAF, and NEMO provides binding platforms for TAK-TAB and IKK activating complexes, leading to NF-*κ*B activation. The DUBs, A20, CYLD, USP2, USP4, USP7, USP10, USP18, USP21, and USP25 terminate NF-*κ*B signaling by removing K63-linked ubiquitin chains from signaling molecules [101–123]. Furthermore, A20, CYLD, and USP25 remove K63-linked ubiquitin chains from TRAF2, TRAF3, and TRAF6. These chains serve as docking platforms for downstream effectors, thereby preventing TRAF2, TRAF3, and TRAF6 from associating with their interaction partners [101, 106, 108, 109, 112, 119–121].

## 5. TLR-Activated Inflammatory Responses and Tumor Progression

Human TLRs are expressed in various immune cells, including dendritic cells, macrophages, monocytes, natural killer cells, B cells, and T cells. In addition, TLRs are often expressed in tumor cells. Tumors, including lung, breast, liver, colorectal, prostate, pancreatic, melanoma, glioma, and esophageal cancers, have elevated TLR expression [124–128] (Table 4). TLRs can be activated in cells by PAMPs of carcinogenic microbes. The best known microbe-related cancers in humans are cervical cancer and oral cancer caused by the human papilloma viruses [129], gastric cancers caused by* Helicobacter pylori* [130], and hepatic cancers caused by hepatitis B and C viruses [131]. In addition, TLRs in tumor cells can be activated by DAMPs, such as HMGB1, S100, and HSPs, released from dying cells following chemotherapy or radiotherapy [132].

TLR activation increases transcription due to the activation of different transcription factors, including NF-*κ*B, AP-1, and IRFs (Figure 1). NF-*κ*B activates the transcription of multiple proinflammatory genes and is a key mediator of acute and chronic inflammatory responses [2–4, 8]. The genes of proinflammatory cytokines TNF-*α* and IL-1*β* are targets of NF-*κ*B, and these cytokines can activate NF-*κ*B signaling as effectively as TLR ligands. TNF-*α* and IL-1*β* are released into the tumor environment when TLRs are activated in tumor cells. This in turn activates NF-*κ*B, resulting in sustained inflammatory cellular responses and chronic inflammation in the tumor microenvironment [6, 7]. In addition, NF-*κ*B also controls the expression of genes involved in cell growth, proliferation, antiapoptosis, angiogenesis, tissue invasion, and metastasis. Typically, NF-*κ*B controls cell growth and proliferation by increasing c-myc and cyclin D1, cyclin D2, cyclin D3, cyclin E, and CDK2, all of which regulate cell cycle progression. It also promotes growth by producing growth factors, including IL-2, IL-6, GM-CSF, and CD40L [133–138]. NF-*κ*B inhibits apoptosis by regulating the antiapoptosis proteins cIAPs, c-FLP, and members of the Bcl-2 family [139]. NF-*κ*B activation increases angiogenesis in tumors. Tumor cell invasion and metastasis are promoted by the upregulation of angiogenic factors, such as vascular endothelial growth factor; metastasis proteins, such as matrix metalloproteinases, urokinase-type plasminogen activator, MCP-1, MIP-1, and cathepsin B; and chemokines, such as IL-8 and CXCL1 in the tumor microenvironment. In addition, the expression of adhesion molecules, such as ICAM-1 and E-selection, is increased in tumor cells [9–11]. Consequently, TLR-activated inflammatory responses promote all stages of tumor progression from tumorigenesis to invasion and metastasis.

Consistent with this, many studies have demonstrated that TLR drives tumor progression. In animal models of HCC and head and neck carcinoma, TLR2 expression in tumor cells correlates positively with tumor progression [140–142]. TLR2 activation in host nontumor cells also has protumor effects. TLR2 depletion in the host cells of mice was reported to reduce the progression of breast, gastric, and intestinal tumors [143, 144]. The proposed mechanism involved TLR2-derived proinflammatory responses [140]. Similarly, protumor functions were observed following TLR4 activation in tumor cells and nontumor host cells. TLR4 activation in tumor cells promoted the growth of breast, lung, head and neck, and liver tumors in animal models [140, 145–149]. TLR4 activation in nontumor host cells increased the development of colitis-associated colorectal tumors and diethylnitrosamine-induced liver cancer in animal models [150, 151]. In addition, TLR5, TLR7, and TLR9 activation promoted tumor growth in different animal models of cancer [152–155]. The promotion of tumor development by TLRs has been supported by clinical data. TLR3 expression in tumor cells has been associated with poor clinical outcome in patients with prostate carcinoma [156]. TLR4 expression in breast and colorectal tumors has also been associated with a poor clinical outcome [157–159]. Strong TLR7 expression was associated with poor prognosis in patients with non-small cell lung cancer and predicted chemotherapy resistance in these patients [160]. Similarly, several studies have revealed an association between TLR9 overexpression in tumor cells and poor prognosis in patients with prostate carcinoma and glioblastoma. TLR9 overexpression has also been associated with an increased tumor grade in breast and ovarian cancers [156, 161, 162].

In contrast to these protumor effects, some studies have shown antitumor effects of TLR activation. TLR signaling elicited antitumor responses in the immune cells of tumor-bearing hosts to facilitate eradication of tumor cells. These results are not discussed in detail here but have been extensively reviewed elsewhere [163–165].

## 6. TLR Activation Enhances Stemness in Cancer Cells

CSCs are a small population of cancer cells found within tumors with high stemness. They are considered to be the cells of origin for tumor initiation and key drivers of malignancy. To achieve this, CSCs exhibit four main properties: (1) capacity to drive neoplastic proliferation and initiate tumors; (2) unlimited capacity for self-renewal; (3) potential to generate more differentiated progeny for heterogeneous cancer cell lineages, and (4) increased resistance to radiation and chemotherapy [12–16]. CSCs can evolve from normal stem cells, which have a long lifespan and are prone to accumulating mutations. Alternatively, CSCs can arise from restricted progenitor or differentiated cancer cells by genetic or epigenetic alterations that activate self-renewal mechanisms and promote stemness [13–16]. CSCs are usually distinguished from other cancer cells by the expression of specific surface markers, including CD133, CD44, CD24, and ALDH. Tumor forming capability can be examined in vitro by analyzing sphere formation in component-defined stem cell medium and low adherence plates. The ability to pump out drugs can be measured by side population analysis using flow cytometry. The tumorigenesis of sorted or enriched CSCs can be investigated using xenograft transplants [12–16].

CSCs play a key role in tumor development, and TLR activation promotes tumor progression; therefore, it is logical to propose that TLR signaling enhances stemness in cancer cells. Emerging evidence has demonstrated an association between NF-*κ*B activation by TLR signaling and the expansion, invasion, and tumorigenesis of CSCs. For example, the TLR2-MyD88-NF-*κ*B signaling pathway supports a proinflammatory microenvironment together with the expansion of the CD44^+^/MyD88^+^ epithelial ovarian cancer (EOC) stem cells by enhancing self-renewal, as shown by the upregulation of stemness-associated genes. CD44^+^/MyD88^+^ EOC stem cells are responsible for therapeutic resistance and recurrence in patients with EOC [166]. The stimulation of breast cancer cells with the TLR3 ligand poly(I:C) enhanced stemness in cancer cells through the simultaneous activation of *β*-catenin and NF-*κ*B signaling pathways. TLR3 activation promoted the expression of stemness-associated genes, including OCT3/4, NANOG, and SOX2 [167]. Increased stem-like properties were associated with TLR4 expression in HCC, and TLR4 expression in HCC cells correlated significantly with enhanced invasion and migration to the splenic vein in nude mice. In clinical HCC tissues, high TLR4 expression correlated strongly with early recurrence and poor survival, which contributed to poor prognosis of HCC [168]. NF-*κ*B suppression by I*κ*B*α*SR in mammary epithelial cells impaired tumorigenesis and diminished tumor-associated macrophage and tumor neoangiogenesis in breast cancer. I*κ*B*α*SR reduced the number of CD44^+^/CD24^−^ stem cells and suppressed NANOG and SOX2 expression. These results indicate that the NF-*κ*B pathway controls the tumorigenesis through regulation of stemness of breast cancer cells [169]. TLR4 activation may also activate TWIST1 and promote the formation of stem-like cancer cells in the mouse liver via a cooperation with nanog and STAT3 [170]. TLR9 expanded stem-like androgen-independent prostate cancer cells through NF-*κ*B and STAT3 activation, which in turn upregulated the expression of stemness-associated genes, including NKX3.1, KLF-4, BMI-1, and COL1A1 [153].

## 7. Interplay between Inflammation and Stemness in Cancer Cells

NF-*κ*B is activated by IL-1*β* and TNF-*α*, which are downstream effectors of TLR activation that also increase stemness in cancer cells. IL-1*β* and TGF-*β* cooperatively upregulate stemness-associated genes, including NESTIN, BMI-1, NOTCH-2, and LIF in glioma cells, thereby increasing invasiveness, drug resistance, and tumor growth in vivo [171]. IL-1*β* stimulated the expression of the stemness-associated genes NESTIN and BMI-1 in colon cancer cells, promoting sphere formation and increasing drug resistance. The expression of the EMT activator ZEB1 increased in IL-1*β*-induced sphere cells, suggesting a connection between EMT and IL-1*β*-induced CSC self-renewal [172]. In the study, TNF-*α* upregulated SLUG expression through canonical NF-*κ*B/HIF1*α* signaling in human breast cancer cells. SLUG upregulation was reported to promote stemness in breast cancer cells, with increased CD44 and Jagged-1 expression, mammosphere growth, and extracellular matrix invasiveness [173]. These findings indicate that NF-*κ*B-mediated inflammatory responses trigged by TLR, IL-1, and TNF-*α* signaling promote stemness in cancer cells.

In contrast, NF-*κ*B-derived inflammatory responses are high in stemness-enriched cancer cells. In acute myelogenous leukemia, a subpopulation of CD34^+^ stemness-enriched cells was reported to exhibit high NF-*κ*B activity, which was not seen in normal hematopoietic stem cells and leukemia cells. This study also identified a TNF-*α* autocrine pathway forming a NF-*κ*B/TNF-*α* positive feedback loop that maintained NF-*κ*B activation [174]. TRA-1-60-, CD151-, and CD166-positive stemness-enriched cancer cells purified from human prostate tumors exhibited increased NF-*κ*B activity and inflammatory gene expression. These cells recapitulated parent tumor heterogeneity in serial xenograft experiments, indicating a hierarchy of human prostate cancer cell development and elevated NF-*κ*B activity. This may represent a functional pathway of stemness-enriched cancer cells in human prostate cancer [22]. NF-*κ*B activation is increased in glioblastoma CSCs. p65/RelA translocation into nuclei is higher in these cells than in non-CSCs [21]. A similar phenomenon was observed in ovarian cancer and breast CSCs. NF-*κ*B activation and cytokine expression were elevated in ovarian CSCs. These stemness-enriched ovarian cancer cells were more resistant to anticancer drugs and were more metastatic than non-CSCs [175]. Stemness-enriched breast cancer cells have a CD44^+^/CD24^−^ phenotype and higher NF-*κ*B activity than CD44^−^/CD24^+^ breast cancer cells [176, 177].

These results indicate a bidirectional interplay between inflammation and stemness in cancer cells. The activation of NF-*κ*B-mediated inflammation by extrinsic stimuli, such as TLR ligands, IL-1, and TNF-*α*, induces stemness in cancer cells. The expression of stemness-associated genes is regulated by NF-*κ*B alone or in cooperation with other signaling pathways, such as STAT3 and NOTCH signaling pathways [18, 19, 21]. The regulation of EMT genes, such as SNAIL, SLUG, and TWIST1, promotes an EMT phenotype, which can initiate the metastasis and dedifferentiation of cancer cells into CSCs [18, 19, 173]. NANOG, SOX2, and POU5F1 are also regulated and are essential for maintaining the pluripotency of CSCs [169, 178–180]. Conversely, increased stemness enhances NF-*κ*B activation in cancer cells. This may involve the intrinsic dysregulation of inflammatory signaling pathways in cancer cells.

## 8. Intrinsically Altered TLR Signaling Enhances Inflammation during the Enrichment of Stemness in Cancer Cells

This concept was illustrated by a recent report that demonstrated the downregulation of negative regulators of TLR signaling when stemness was enriched in head and neck squamous cell carcinoma (HNSCC) cells [181]. These downregulated negative regulators include COMMD1, SOCS1, and PDLIM2 control TLR signaling through ubiquitination and proteasomal degradation (Figure 1 and Table 2). COMMD1 was initially described as mouse U2af1-rs1 region 1 (Murr1) because it was first discovered in close proximity to U2af1-rs1 [182]. This protein lacks catalytic activities and was hypothesized to be an adaptor or scaffold protein with a role in protein-protein interactions [183, 184]. COMMD1 interacts with the p65/RelA subunit of NF-*κ*B to promote ubiquitination and proteasomal degradation of the subunit [96, 97, 185]. SOCS1 acts as a ubiquitin ligase through its SOCS box domain leading to ubiquitination and proteasomal degradation of p65/RelA. Thus, SOCS1 restricts prolonged p65/RelA transactivation and terminates NF-*κ*B-mediated inflammatory gene expression [98, 186]. In addition, TLR activation induces the expression of SOCS1, which is phosphorylated by Bruton's tyrosine kinase, and then targets TIRAP for proteasomal degradation [95]. SOCS1 also interacts with IRAK to attenuate TLR signaling [187]. PDLIM2 functions as E3 ubiquitin ligase through its LIM domain and facilitates the polyubiquitination of p65/RelA. PDLIM2 binds p65/RelA through its PDZ domain, sequesters soluble p65/RelA within promyelocytic leukemia protein bodies, and subsequently assists the proteasomal degradation of p65 [181].

COMMD1 was downregulated by miR-205 during the enrichment of stemness in HNSCC cells. The reversal of COMMD1 downregulation correlated with the upregulation of the expression of NF-*κ*B-controlled inflammatory genes, including PTGS2, IL8, IL-1A, CXCL2, IL-6, STAT5B, STAT3, CCL2, IL-1B, CD40, and IL-15. COMMD1 downregulation in cancer cells induced NF-*κ*B activation and inflammatory responses in cancer cells and the tumor microenvironment. In addition, stemness was increased by reduced COMMD1 expression, as shown by increased sphere-forming capability and elevated expression of stemness-associated genes. COMMD1 downregulation promoted tumorigenicity and tumor growth and increased inflammation and stemness. Furthermore, COMMD1 downregulation was shown in head and neck, breast, lung, colon, gastric, and prostate cancers [181]. These findings indicate that altered TLR signaling drives a positive feedback interplay between inflammation and stemness in cancer cells.

## 9. Conclusion

Small CSC populations in tumors are key players in malignancy and reduce the success of cancer therapies. Most tumor tissue is removed by chemotherapy, radiotherapy, and surgery, but relapses occur if CSCs remain. Thus, understanding the underlying molecular mechanisms of CSC expansion is crucial for identifying new cancer therapies.

Chronic inflammation has been identified as a major factor for CSC expansion and tumor progression. As reviewed in this article, chronic inflammation can be initiated in the tumor microenvironment by an extrinsic cellular pathway in which TLRs are stimulated by PAMPs of carcinogenic microbes and DAMPs released from cancer cells killed during anticancer treatments. This activates NF-*κ*B-mediated inflammation and increased stemness in cancer cells. Conversely, altered TLR signaling can lead to persistent activation of NF-kB-mediated inflammation during the enrichment of stemness in cancer cells.

This bidirectional positive feedback loop of inflammation and stemness in cancer cells can be a mechanism underlying malignancy and reduced treatment success. Blocking this loop by targeting TLR signaling may represent an effective strategy for inhibiting CSC expansion and tumor progression.

## Figures and Tables

**Table 1:** TLRs, their cellular location, ligand recognitions, and adaptor usage.

Type of TLR	Cellular location	Exogenous ligands (PAMP)	Endogenous ligands (DAMP)	Signal adaptor
TLR1 (in association with TLR2)	Cell surface	Bacteria: triacyl lipopeptides	Unknown	MyD88
TLR2 (in association with TLR1 or TLR6)	Cell surface	Bacteria: peptidoglycan, lipoproteins, lipoteichoic acid, lipoarabinomannan, glycophosphatidylinositol anchors, porin; fungi: zymosan	HSP60, HSP70, Gp96 biglycan, hyaluronic acid, hyaluronan, HMGB1, versican, monosodium urate crystal	MyD88/TIRAP
TLR6 (in association with TLR2)	Cell surface	Mycoplasma: macrophage-activating lipopeptide 2	Versican	MyD88
TLR3	Endosomal compartment	Viruses: dsRNA	mRNA	TRIF
TLR4	Cell surface	Bacteria: LPS Viruses: RSV fusion protein Fungi: mannan Protozoa: glycoinositolphospholipids	HSP22, HSP 60, HSP70, HSP72, Gp96, HMGB1, S100, oxidized phospholipids, heparin sulfate, fibrinogen, fibronectin, tenascin-C, b-defensin 2, versican, hyaluronic acid, hyaluronan	MyD88/TIRAP/ TRAM/TRIF
TLR5	Cell surface	Bacteria: flagellin	Unknown	MyD88
TLR7	Endosomal compartment	Viruses: ssRNA	ssRNA (immune complex)	MyD88
TLR8	Endosomal compartment	Viruses: ssRNA	ssRNA (immune complex)	MyD88
TLR9	Endosomal compartment	Bacteria: CpG DNA Viruses: CpG DNA Protozoa: CpG DNA, haemozoin	Chromatin IgG complex, HMGB	MyD88

**Table 2:** Negative regulators of TLR signaling involved in ubiquitination.

E3 ligase or adapter of E3 ligase complex	Target molecules	Ubiquitin-mediated modifications	Biological function
A20	RIP1, Ubc13	K48	Proteolytic degradation
Triad3A/RNF216	TLR3, TLR4, TLR5, TLR9, TIRAP, TRIP, RIP1	K48	Proteolytic degradation
SOCS1	TIRAP, IRAK, p65/RelA	K48	Proteolytic degradation
PDLIM2	P65/RelA	K48	Proteolytic degradation
COMMD1	P65/RelA	K48	Proteolytic degradation
TRIM27	IKK*α*, IKK*β*	K48	Proteolytic degradation
TRIM38	TRAF6, TRIF, TAB2/3	K48	Proteolytic degradation

**Table 3:** Negative regulators of TLR signaling involved in deubiquitination.

dUb	Target molecules	Ubiquitin-mediated modifications	Biological function
A20	RIP1, RIP2, TRAF2, TRAF6, MALT1, NEMO	K63	Signaling termination
CYLD	MyD88, TRAF2, TRAF6, TRAF7, RIP1, NEMO	K63	Signaling termination
USP2*α*	TRAF6	K63	Signaling termination
USP4	TRAF2, TRAF6, TAK1	K63	Signaling termination
USP7	TRAF6, NEMO	K63	Signaling termination
USP10	TRAF6, NEMO	K63/M1	Signaling termination
USP11	I*κ*B*α*	K48	Proteolytic degradation
USP15	I*κ*B*α*	K48	Proteolytic degradation
USP18	TAK1, NEMO	K63	Signaling termination
USP21	RIP1	K63	Signaling termination
USP25	TRAF2, TRAF3, TRAF5, TRAF6	K63/K48	Signaling termination/proteolytic degradation

**Table 4:** TLR expression profile.

Type of TLR	Immune cells	Tumor cells
TLR1	cDCs, eosinophils, monocytes, neutrophils, NK cells, pDCs, B cells	Myeloma cells
TLR2	cDCs, monocytes, neutrophils, NK cells, B cells, T cells	Breast cancer, gastric carcinoma, HCC, intestinal carcinoma, laryngeal carcinoma, myelogenous leukemia, oral squamous cell carcinoma
TLR3	cDCs, NK cells	Breast cancer, cervical cancer, CRC, esophageal squamous cell carcinoma, gastric carcinoma, HNSCC, HCC, laryngeal carcinoma, lung carcinoma, melanoma, myelogenous leukemia, neuroblastoma cells, ovarian cancer, pharyngeal carcinoma (cell lines), prostate cancer
TLR4	cDCs, eosinophils, monocytes, neutrophils	Adrenocortical carcinoma, breast cancer, cervical cancer, CRC, epithelial ovarian cancer, esophageal squamous cell carcinoma, gastric carcinoma, HNSCC, intestinal carcinoma, laryngeal carcinoma, HCC, lung carcinoma, melanoma cell lines, myelogenous leukemia, neuroblastoma, ovarian cancer, pancreatic cancer, prostate cancer, skin cancer
TLR5	cDCs, monocytes, neutrophils, NK cells, T cells	Breast cancer cells, cervical squamous cell carcinoma, CRC, gastric carcinoma, intestinal carcinoma, ovarian cancer
TLR6	cDCs, monocytes, neutrophils, NK cells, B cells	
TLR7	Eosinophils, monocytes, neutrophils, pDCs, B cells	CRC, esophageal squamous cell carcinoma, lung carcinoma, myeloma cells, pancreatic ductal adenocarcinoma
TLR8	cDCs, monocytes, neutrophils T cells, Tregs	CRC, lung carcinoma
TLR9	Eosinophils, monocytes, neutrophils, pDCs, B cells	Breast cancer, cervical squamous cell carcinoma, CRC, esophageal squamous cell carcinoma, gastric carcinoma, lung carcinoma, myeloma cells, myelogenous leukemia, ovarian cancer (cell lines), prostate cancer, renal cell carcinoma
TLR10	Eosinophils, monocytes, neutrophils, pDCs, B cells, T cells, Tregs	CRC

HCC, hepatocellular carcinoma; CRC, colorectal carcinoma; NHSCC, head and neck squamous cell carcinoma.
